# Carbon Dot Integrated Cellulose-Based Green-Fluorescent Aerogel for Detection and Removal of Copper Ions in Water

**DOI:** 10.3390/gels11080655

**Published:** 2025-08-18

**Authors:** Guanyan Fu, Chenzhan Peng, Jiangrong Yu, Jiafeng Cao, Shilin Peng, Tian Zhao, Dong Xu

**Affiliations:** 1 Hunan Occupational Disease Prevention and Control Institute, Changsha 410004, China; 18512382807@163.com; 2 National Key Laboratory of Woody Oil Resources Utilization, College of Food Science and Engineering, Central South University of Forestry & Technology, Changsha 410004, China; culaisett@163.com (C.P.); caojiafengc13549555217@163.com (J.C.); 3 School of Materials Science and Engineering, Hunan University of Technology, Zhuzhou 412007, China; 18593328326@163.com (J.Y.); psl18670833816@163.com (S.P.)

**Keywords:** carbon dot, aerogel, copper ion, removal, fluorescent sensing

## Abstract

Industrial pollution caused by Cu(II) ions remains one of the most critical environmental challenges worldwide. A novel green-fluorescent aerogel has been successfully developed for simultaneous sensing and adsorption of Cu(II) through the cross-linking of carboxymethyl nanocellulose and carbon dots (C dots) using epichlorohydrin as a linker. The C dots were synthesized by heating glucose and aspartate mixed solutions at 150 °C. Under UV illumination, the aerogel exhibited intense homogeneous green fluorescence originating from the uniformly dispersed C dots, whose emission can be efficiently quenched by Cu(II) ions. By leveraging smartphone-based imaging, the concentration of Cu(II) was quantified within the range of 5–200 µg/L, with a detection limit of 3.7 µg/L. The adsorption isotherm of Cu(II) onto the aerogel strictly conformed to the Freundlich isotherm model (fitting coefficient R^2^ = 0.9992), indicating a hybrid adsorption mechanism involving both physical adsorption and chemical complexation. The maximum adsorption capacity reached 149.62 mg/g, a value surpassing many reported adsorbents. X-ray photoelectron spectroscopy and Fourier-transform infrared spectroscopy analyses confirmed that the interactions between the aerogel and Cu(II) involved chelation and redox reactions, mediated by functional groups such as hydroxyl, amino, and carboxyl moieties. The straightforward fabrication process of the aerogel, coupled with its low cost, abundant raw materials, facile synthesis, and superior Cu(II) removal efficiency, positions this bifunctional fluorescent material as a promising candidate for large-scale environmental remediation applications.

## 1. Introduction

Copper, a highly versatile metal integral to industrial manufacturing, advanced technology, and biomedical applications, simultaneously poses a significant threat as an environmental contaminant. As an essential trace element, it is vital for human health and plays a key role in various physiological processes such as enzymatic catalysis, immune function, cellular structure maintenance, neuroendocrine regulation, hematopoiesis, oxygen transport etc. [1]. Body growth and maintenance are associated with copper, which are essential for enzymatic reactions, oxidative phosphorylation, and the proper functioning of the central nervous system [2]. Both excessive and deficient levels of Cu(II) ions can be detrimental to health. A normal human contains 100–200 mg of copper, with approximately 60% found in muscles and bones, 20% in the liver, and about 10% in the bloodstream [3]. Despite the relatively small proportion of copper in the body, a deficiency can result in growth and metabolic issues. In cases of bioaccumulation, particularly in the liver of humans and animals, copper is toxic and lead to oxidative stress and various disorders, including neurodegenerative conditions such as Wilson’s disease, Alzheimer’s disease, Parkinson’s disease, and Menke’s disease [4,5,6,7]. Similar to other heavy metals [8], copper is non-biodegradable and can persist in the environment, eventually accumulating and biomagnifying through the food chain over time, posing substantial risks to human safety [9,10]. Numerous countries and organizations have established regulations concerning acceptable levels of copper in drinking water and the allowable concentrations of heavy metals in industrial effluents. For instance, the World Health Organization (WHO) has set a standard for copper concentration in drinking water at 2 mg/L, while the U.S. Environmental Protection Agency (EPA) recommends a limit of 1.3 mg/L [11]. Contaminated water by Cu(II) ions is a primary route of human exposure to this metal [12]. Therefore, detecting and removing Cu(II) ions in water has become a key focus of current research.

Among the various methods available for treating copper-containing wastewater, adsorption methods have garnered significant attention as the most cost-effective option, thanks to their high efficiency and operational simplicity [13]. There are numerous adsorbent materials employed to capture Cu(II) ions, including activated carbon [14,15,16], bentonite [17,18,19], silica [20,21], and graphene [22,23,24]. However, while these materials can effectively adsorb Cu(II) ions, they are often constrained by limited availability of raw materials and high production costs. Cellulose is the most prevalent polysaccharide in nature, constituting over 50% of the carbon content in plants [25,26,27], and has been extensively explored for direct applications in removing heavy metal ions [28,29,30], dyes [31,32,33], and antibiotics [34,35,36]. In recent years, cellulose has been engineered into aerogels [37], which are ultralight and have three-dimensional structures with networks of pores that that exhibit enhanced adsorption performance due to their interconnected pore architecture [38,39]. Cellulose is preferred as a component due to its inherent renewability, abundant surface hydroxyl groups, and facile processability. Nevertheless, there are few reports focusing on its application for Cu(II) ion sorption. Notably, a biodegradable cellulose-based hydrogel has been designed for the adsorption of Cu(II) ions with the highest adsorption capacity of 28.4 mg/g, highlighting the potential of cellulose-based materials in heavy metal remediation [40].

Carbon dots (C dots) are fluorescent carbon-based nanomaterials, characterized as zero-dimensional nanoparticles measuring less than 10 nm in size [41,42,43]. They offer several advantages, including biocompatibility, high brightness, resistance to photoirradiation and salt, and straightforward preparation [44,45,46]. The presence of surface functional groups such as carboxylates and amines facilitates the conjugation of C dots with recognition elements, such as antibodies and aptamers, thus expanding their application scope across diverse fields [47,48,49,50,51,52]. Additionally, C dots are brighter and more stable compared to metal-based fluorescent nanomaterials like gold nanoclusters [53]. Unlike semiconductor quantum dots, which exhibit issues such as photoblinking and photobleaching, many C dots are free from these drawbacks, making them suitable for a wide range of applications in sensing, imaging, and as LED fluorophores [54]. They can also be incorporated into aerogels to endow these materials with fluorescent or catalytic properties.

**Scheme 1:**
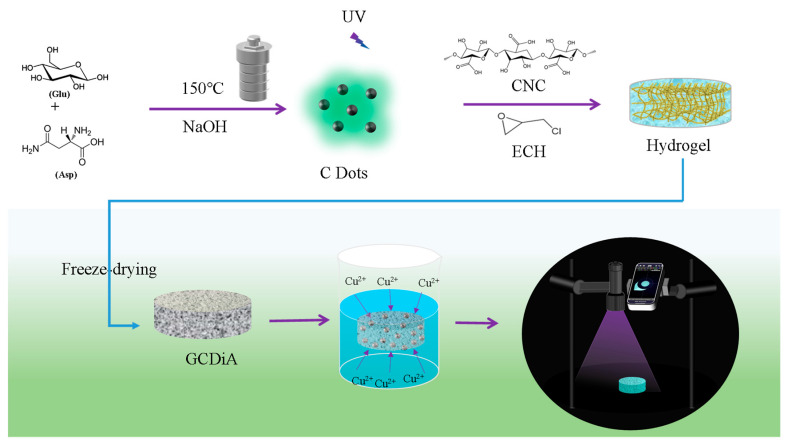
Synthesis of green carbon dot integrated aerogels (GCDiA) via using green-emissive carbon dots and cellulose nanocrystals (CNC) as the bricks and epichlorohydrin (ECH) as the cross-linking agent for efficient absorption and sensing of Cu(II).

This study aims to develop a green-fluorescent carbon dot-integrated aerogel (GCDiA) designed for effective Cu(II) ion sorption and sensing capabilities via smartphone technology. The GCDiA is prepared by simply cross-linking C dots with cellulose nanocrystals using epichlorohydrin (ECH). The C dots were synthesized by heating glucose and glutamine mixed solutions at 150 °C. The strong fluorescence emitted by the C dots can be quenched by Cu(II) ions, allowing for visual monitoring of Cu(II) content under UV light (Scheme 1). The composition of the aerogels is optimized by adjusting the amounts of the components. The adsorption of Cu(II) ions is investigated over a concentration range of 5–1000 mg/L, and the mechanisms underlying Cu(II) ion adsorption onto the aerogel are thoroughly analyzed using X-ray photoelectron spectroscopy and Fourier-transform infrared spectroscopy.

## 2. Results and Discussions

### 2.1. Characterization of the Novel Aerogel

**Figure 1:**
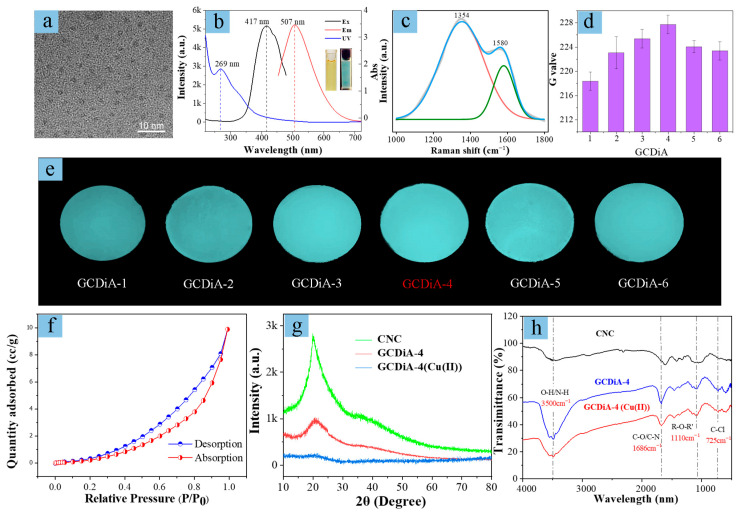
(**a**) TEM images of C dots. (**b**) UV-Vis absorption and fluorescence spectrum of C dots. The inserts are of C dots solution under natural and UV light. (**c**) Raman spectrum. (**d**) Fluorescence performance of different GCDiAs. (**e**)The corresponding fluorescence images of GCDiAs. (**f**) Pore characteristics of GCDiA-4. (**g**) XRD and (**h**) FT-IR spectra of CNC and GCDiA-4 before and after Cu(II) adsorption.

We hydrothermally synthesized Cu(II)-responsive C dots by using glucose and aspartate as the carbon precursors, leveraging their outstanding optical and chemical properties [55]. Under natural light, the C dots solution appears pale yellow, while it fluoresces bright green under ultraviolet light. The TEM images, UV-Vis absorption spectra, and fluorescence spectra of the C dots are shown in Figure 1a,b. These C dots possess a diameter of approximately 1.64 ± 0.09 nm and exhibit high uniformity. A prominent peak at 269 nm is observed, attributed to σ- σ bonds. In the fluorescence spectra, the C dots demonstrate maximum excitation and emission wavelengths at 417 nm and 507 nm, respectively. The Raman spectrum showed that the C dots have a D-band at 1354 cm^−1^ and a G-band at 1580 cm^−1^ (Figure 1c). The intensity ratio (ID/IG) between D and G bands is 1.68, indicating the low graphitization in C dots and more defects and disordered structures in them.

The proportions of C dots and CNC significantly influence the fluorescence of the GCDiA. Among the variations, GCDiA-4 exhibited the strongest fluorescence, making it the selected candidate for Cu(II) sensing and adsorption (Figure 1d,e). Previous studies have indicated that the formation of aerogels can lead to increased quantum yield; however, higher concentrations of C dots and CNC often lead to self-quenching effects. Figure 1f illustrates the N_2_ adsorption-desorption isotherm for C dots, with the inset displaying the pore size distribution curve. The BET surface area and average pore diameter for GCDiA-4 were found to be 8.622 m^2^/g and 5.329 nm, respectively. These findings validate that GCDiA-4 possesses a typical three-dimensional structure with abundant porosity.

X-ray diffraction (XRD) analyses of CNC and GCDiA-4 before and after Cu(II) adsorption are illustrated in Figure 1g. CNC displays a prominent peak at 20.2°, indicating relatively high crystallinity and a likely well-ordered crystal structure, corresponding to the crystalline structure of cellulose [56]. The intensity of this peak diminishes and the peak shape is broadened as a result of the formation of GCDiA-4 by cross-linking between CNC and C dots, indicating a disruption in the cellulose crystallinity. Following Cu(II) adsorption, peak intensity decreases further, proving the disruption of the CNC crystal structure due to interactions between Cu(II) and CNC.

The functional groups present in CNC and GCDiA-4 before and after Cu(II) adsorption were examined using FT-IR spectroscopy, as shown in Figure 1h. Upon aerogel formation, the stretching vibration peaks for O-H and N-H around 3500 cm^−1^ were notably intensified compared to CNC. A distinct peak for C-Cl appeared at 725 cm^−1^, confirming the successful cross-linking between CNC and C dots. After the aerogel GCDiA-4 was formed, a characteristic ether bond peak emerged at 1110 cm^−1^, signifying that the ECH cross-linking was effective. After post-adsorption, the C-O and N-O stretching vibrations underwent significant changes from 1678 cm^−1^ to 1686 cm^−1^, confirming that the adsorption process of Cu(II) involved amino, hydroxyl, and carboxyl groups [57,58].

### 2.2. Cu(II) Adsorption and Detection

**Figure 2:**
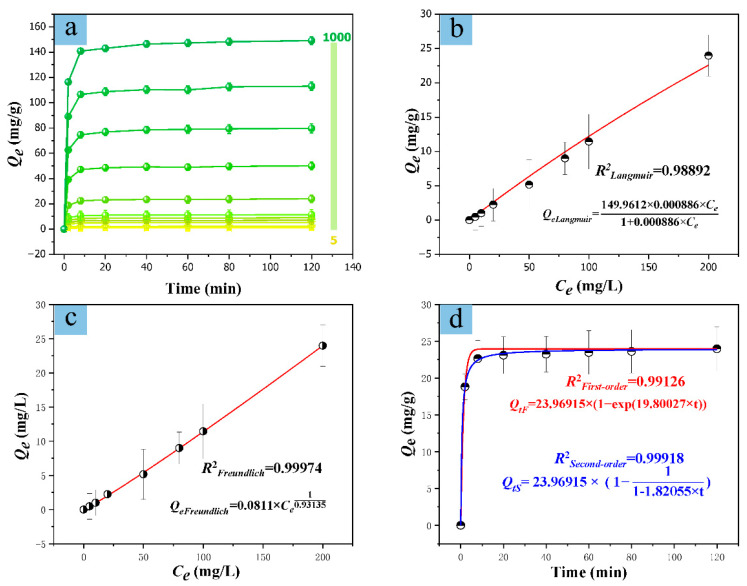
(**a**) Time-dependent adsorption of Cu(II) on GCDiA-4 with its initial concentration over the range of 10–1000 mg/L. (**b**) Langmuir isotherm plot for Cu(II) absorption at 25 °C, where Ce is the equilibrium Cu(II) concentration in the solution. (**c**) Freundlich isotherm plot for Cu(II) absorption at 25 °C. (**d**) Pseudo-first order and pseudo-second order kinetic model for Cu(II) adsorption.

To explore the adsorption kinetics, the time-dependent uptake of Cu(II) by GCDiA-4 across an initial concentration range of 5–1000 mg/L is illustrated in Figure 2a. The results clearly show that the adsorption process was rapid, with over 95% of Cu(II) in the solution adsorbed by GCDiA within just 10 min of immersing the aerogel. By 40 min, the adsorption approached its maximum capacity. This swift sorption can be attributed to the strong interactions between GCDiA and Cu(II), facilitated by functional groups such as -NH_2_. Additionally, the unique porous three-dimensional structure of GCDiA contributes to this efficiency by providing a significant surface-to-volume ratio, which enhances the availability of functional groups for Cu(II) binding.

The initial concentration of Cu(II) significantly influences the adsorption capacity of the aerogels. Typically, a higher concentration of Cu(II) correlates with increased adsorption in the aerogels. With Cu(II) concentration of 1000 mg/L, the adsorption capacity reached 149.12 mg/g. To analyze the adsorption isotherms, both the Langmuir model (Figure 2b) and Freundlich model were applied (Figure 2c). The amount of adsorbed Cu(II) (Qe) is contingent upon the equilibrium concentration (Ce) at 25 °C. According to the Langmuir model, the maximum adsorption capacity for Cu(II) was calculated to be 149.62 mg/g, indicating a high adsorption ability (see Table 1).

The coefficients (R^2^) suggest that the Freundlich model better fits the adsorption isotherms. These findings imply that the adsorption of Cu(II) by the aerogel does not occur as a monolayer but rather involves both physical and chemical processes. To further elucidate the adsorption mechanism, both pseudo-first-order and pseudo-second-order models were employed (Figure 2d). The R^2^ value for the pseudo-second-order model was more favorable than that of the pseudo-first-order model (0.9992), providing additional evidence that the adsorption of Cu(II) on GCDiA-4 involves both physical and chemical interactions.

### 2.3. Selectivity and Sensitivity of GCDiA-4 Toward Cu(II)

**Figure 3:**
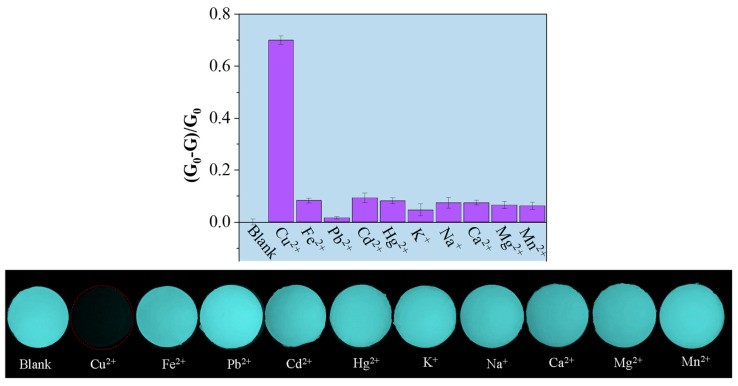
The selectivity of the GCDiA-4 toward Cu(II) over various metal ions including Fe^2+^, Pb^2+^, Cd^2+^, Hg^2+^, K^+^, Na^+^, Ca^2+^, Mg^2+^, and Mn^2+^. The images (**top**) and histogram of (G_0_ − G)/G_0_ (**bottom**).

We examined the selectivity and sensitivity of the GCDiA-4 aerogel for sensing Cu(II) ions. The aerogel was individually immersed in various metal ion solutions, including Fe^2+^, Pb^2+^, Cd^2+^, Hg^2+^, K^+^, Na^+^, Ca^2+^, Mg^2+^, and Mn^2+^ at 1000 mg/L, respectively (Figure 3). Notably, only Cu(II) significantly quenched the fluorescence, while the other ions do not induce substantial color changes. The distinct Cu^2+^-triggered fluorescent quenching thus results from two synergistic mechanisms: selective quenching of C dots by Cu^2+^ and their preferential adsorption of Cu^2+^. While the GCDiA-4, with hydroxyl (-OH) and carboxyl (-COOH) groups, non-specifically adsorbs other metal ions to some extent, this adsorption is much weaker than its affinity for Cu^2+^ because of the chemical groups in C dots. The carbonization of D-glucose and L-aspartate as precursors leads to the formation of specific surface functional groups, which are arranged in a configuration that creates selective binding sites for Cu(II) ions [69]. Therefore, the adsorption and the fluorescence quenching is selective for Cu(II).

**Figure 4:**
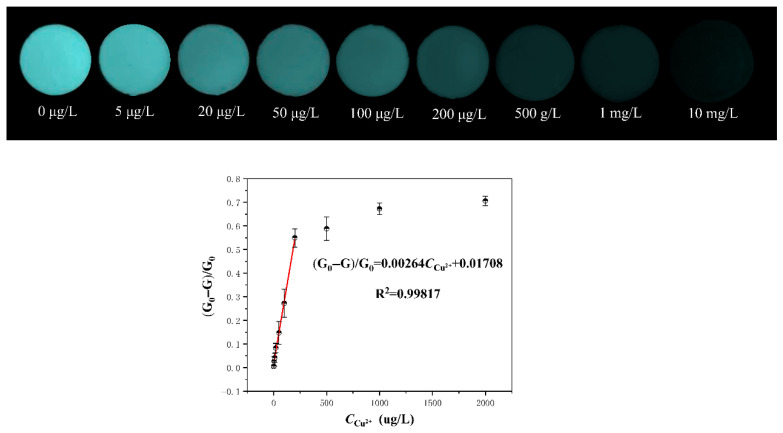
The images and (G_0_ − G)/G_0_ of GCDiA-4 with the presence of 0–10 mg/L Cu(II).

To assess the sensitivity of GCDiA-4 towards Cu(II), the aerogel was exposed to Cu(II) solutions with concentrations ranging from 0 to 10 mg/L. The images and corresponding fluorescence intensity ratios (G_0_ − G)/G_0_ of these aerogels, recorded using the Colorimeter app, are displayed in Figure 4. As the concentration of Cu(II) increased, GCDiA-4 exhibited significant color changes from green to black. The ratio (G_0_ − G)/G_0_ also increased proportionally. A linear relationship was established (G_0_ − G)/G_0_ and Cu(II) concentration within the range of 5–200 µg/L, characterized by the regression equation (G_0_ − G)/G_0_ = 0.00264C_Cu_ + 0.01708 and an R^2^ value of 0.998. The detection limit was determined to be 3.7 µg/L.

These findings indicate that GCDiA-4 not only serves as an effective adsorbent for Cu(II) but also demonstrates high sensitivity and selectivity towards this ion. This capability enhances the practicality of these aerogels for real-world applications, allowing for both the assessment of initial concentrations in wastewater and the determination of whether the adsorption process has reached equilibrium. Moreover, the fabrication of these aerogels is straightforward, and the materials used are cost-effective, facilitating large-scale production and practical use. Large-scale hydrothermal synthesis of C dots is a crucial procedure. Their sustainable and efficient production can be achieved through two key process optimizations. (I) Continuous production via optimized pipeline systems. Similar to beverage pasteurization technologies, a continuous flow pipeline with strategic bends is used. By regulating the precursor flow rate, the heating residence time for C dots formation is precisely controlled. (II) Green synthesis through water vapor heating and heat recovery. Leveraging the moderate reaction temperature (150 °C), water vapor serves as a safe indirect heat source, avoiding fire hazards from direct flame/electrical heating while ensuring uniform temperature. Integrated heat recovery systems reuse waste heat from cooling, lowering energy consumption (aligning with sustainability goals), reducing equipment thermal stress, extending lifespan, and enhancing operational safety.

### 2.4. Mechanism Analysis

**Figure 5:**
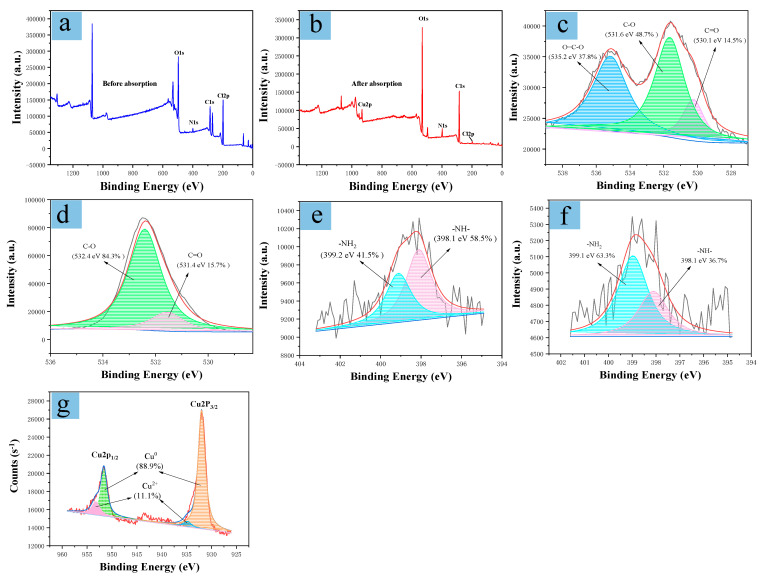
Full XPS spectrum (**a**,**b**) and high-resolution XPS spectra of O 1s (**c**,**d**), N 1s (**e**,**f**) of GCDiA-4 before (**a**,**c**,**e**) and after (**b**,**d**,**f**) Cu(II) absorption. High-resolution XPS spectrum of Cu 2p (**g**) of GCDiA-4 after Cu(II) adsorption.

In Figure 5, peaks for C 1s (284.8 eV), N 1s (400.2 eV), O 1s (499.4 eV), and Cl 2p (199.6 eV) were detected, indicating that the aerogel consists of C, N, O, and Cl. The presence of Cl 2p is due to the crosslinking reagent ECH. Figure 5b shows the full XPS spectral scan of GCDiA-4 after Cu(II) adsorption, where two new peaks at 932 eV and 952 eV represent the Cu 2p1/2 and Cu 2p2/3 orbitals of Cu. Figure 5c,d display the XPS spectra of O1s in GCDiA-4 before and after Cu(II) adsorption. Before adsorption, peaks at 535.2 eV and 531.6 eV correspond to O-C=O and C-O, with a small peak for C=O at 530.1 eV. After adsorption, O-C=O is nearly absent, while the ratio of C=O remains largely unchanged, a phenomenon not observed in previous studies. This is obviously due to the strong interactions between O-C=O with Cu(II) such as complexation. The N 1s peaks in GCDiA-4 (Figure 5e,f) show significant changes in the -NH_2_/-NH ratio before and after adsorption, with more nitrogen detected as -NH_2_ after adsorption, suggesting that part of the -NH- has been converted to -NH_2_ during Cu(II) adsorption, which may be due to the formation of -NH-Cu(II). After Cu(II) adsorption, Figure 5g reveals that Cu^0^ was found in the GCDiA-4, comprising 88.9% of the total, while Cu(II) decreased to 11.1%, indicating that they were captured and reduced by GCDiA-4. We propose the following mechanism for the rapid adsorption and sensing of Cu(II): (i) GCDiA-4 quickly adsorbs Cu(II) upon contact due to its 3D porous structure and physical interactions. (ii) The O=C-O and -NH- groups interact strongly with Cu(II). (iii) The aerogel can reduce Cu(II) to Cu0 after adsorption. (iv) Cellulose aids in Cu(II) adsorption in GCDiA-4, while nitrogen carbon dots facilitate both adsorption and reduction of Cu(II). This is the reason why the crystallinity of GCDiA-4 was further disrupted in XRD data. Consequently, Cu(II) concentration can be quantified through fluorescence quenching once adsorption equilibrium is achieved. In summary, the sensing and effective absorption of Cu(II) involve both physical and chemical processes.

### 2.5. Electroplating Chrome Wastewater Adsorption

**Figure 6:**
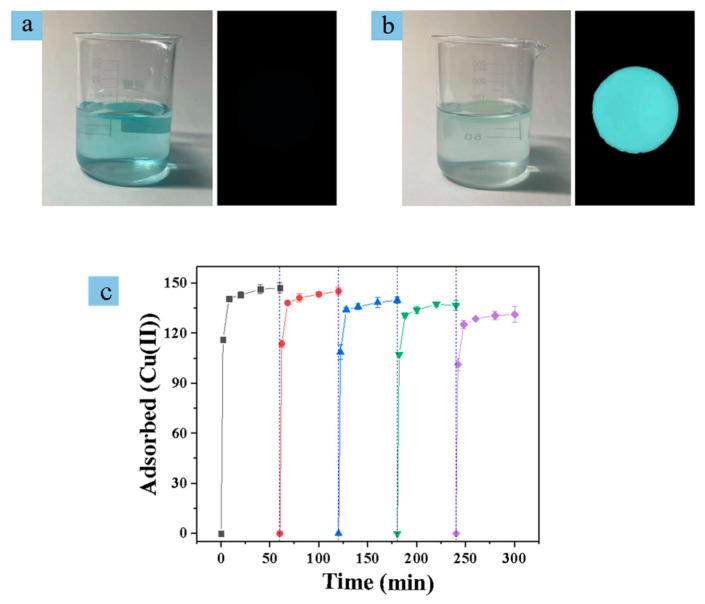
(**a**,**b**) The photograph of the Cu(II) contaminated wastewater (left) and the fluorescence of the aerogel after adsorption (right) both initially (**a**) and after treatment with five aerogels (**b**). (**c**) Temporal evolution of Cu(II) adsorption capacity by the aerogel in five cyclic adsorption–desorption tests.

The original cleaning wastewater from printed circuit boards appears as a clear bright blue after filtration. Upon adding GCDiA-4 to adsorb Cu(II), the aerogel exhibits almost no fluorescence under UV light (Figure 6a), making it impossible to quantify the concentration due to its high levels. Following 5-round adsorption, the solution color significantly lightens to a faint blue (Figure 6b), indicating a considerable reduction in Cu(II) concentration. Standard curve analysis reveals that the Cu(II) content in the treated sample is less than or equal to 5 μg/L. It is below the copper content limit established in GB 8978-1996 for wastewater discharge, thereby demonstrating its potential for effectively treating and monitoring Cu(II) contaminated wastewater from printed circuit boards. The regenerative performance of the aerogels, a critical attribute for practical applications, was systematically evaluated. For GCDiA-4, its Cu(II) adsorption capacity could be effectively restored by immersing the used aerogels in a mixed desorption solution consisting of 0.2 M HNO_3_, 0.1 M HCl, and 0.05 M ethylenediaminetetraacetic acid (EDTA) for 6 h. HNO_3_ and HCl can efficiently convert copper species into soluble Cu(II), while EDTA acts as a strong chelating agent for Cu(II). Their synergistic effect ensures effective regeneration of the aerogels. Notably, the fluorescence property of GCDiA-4 was recovered after regeneration process, demonstrating excellent regenerative efficiency. The results showed that the sorption efficiency decreased by only 1.4% after the first adsorption cycle, and the total efficiency loss increased to 9.9% following five sorption-desorption cycles (Figure 6c). These results indicate that GCDiAs exhibit promising reusability as sorbents for Cu(II) removal and are highly useful for copper recycling.

## 3. Conclusions

We developed a novel aerogel, GCDiA, exhibiting heterogeneous green-emissive fluorescence that is effective for both sensing and adsorbing Cu(II). This aerogel is created by cross-linking two nanomaterials, CNC and C dots, using ECH as the cross-linking molecules. GCDiA demonstrated a remarkable adsorption capacity for Cu(II), reaching a maximum of 149.62 mg/g, and its adsorption behaviour conformed to pseudo-second-order kinetics and Freundlich isotherm models. By capturing fluorescence images via a smartphone, we effectively quantified Cu(II) concentrations between 5–200 μg/L. The significant Cu(II) adsorption results from synergistic physical and chemical interactions, with the fluorescence quenching of GCDiA attributed to the interaction of Cu(II) with the C dots. This innovative functional aerogel shows promising potential for real-world applications in treating Cu(II)-contaminated water, allowing for Cu(II) concentration evaluation and in-situ signaling of aerogel replacement timing. By extending this design strategy, a variety of new functional aerogels can be developed for broader environmental remediation applications.

## 4. Experimental

### 4.1. Chemicals

Avicel PH-101 microcrystalline cellulose (50 µm), D-glucose (99.8%), L-aspartate (96%), potassium dichromate (K_2_Cr_2_O_7_, 99%), potassium chloride (KCl, 99%), sodium chloride (NaCl, 99%), calcium chloride dihydrate (CaCl_2_·2H_2_O, 99.7%), nickel chloride (NiCl_2_, 99%), cadmium chloride (CdCl_2_, 99%), nickel chloride (NiCl_2_, 99%), ferric chloride (FeCl_3_), magnesium sulphate (MgSO_4_, 99%), mercury sulphate (HgSO_4_, 98%), cobalt sulphate heptahydrate (CoSO_4_·7H_2_O, 98%), manganese sulphate (MnSO_4_, 98%), ECH, sodium hydroxide (NaOH, 99%), sodium bromide (NaBr, 99%), and sodium hypochlorite (NaClO) were purchased from Sinopharm Group Co., Ltd. (Shanghai, China). 2,2,6,6-tetramethylpiperidine 1-oxyl (TEMPO) was bought from Macklin (Shanghai, China). Double distilled water (DD) was prepared by a MILLI-Q^®^ HX 7000 Millipore water purification system (Merck Investment Co., Ltd., Shanghai, China) and utilized in all the experiments. All glassware used in the experiments was washed in freshly prepared aqua regia (HCl:HNO_3_ volume ratio = 3:1) and rinsed thoroughly with ultrapure water before use.

### 4.2. Characterizations

The surface area and porosity of the aerogels were measured using an Autosorb-iQ gas adsorption analyser from Quantachrome Instruments (Boynton Beach, FL, USA). The morphology of the green C dots and the aerogels was observed using a Helios NanoLab G3 UC scanning electron microscope (SEM) and a FEI Tecnai G F20 transmission electron microscope (TEM), both from ThermoFisher (Waltham, MA, USA). Crystal structure analysis was conducted using a Bruker D8 X-ray diffractometer (XRD, Billerica, MA, USA). Fourier transform infrared (FT-IR) spectra were obtained using an IN 10™ Fourier transform infrared spectrometer (Nicolet, ThermoFisher, Waltham, MA, USA). X-ray photoelectron spectroscopy (XPS) measurements were performed with a Scientific K-Alpha photoelectron spectrometer (ThermoFisher, Waltham, MA, USA). An atomic fluorescence spectrometer (AFS, AFS-8800, BJHG, Hangzhou, China) and a Shimadzu UV-2550 ultraviolet-visible (UV-Vis) spectrometer (Kyoto, Japan) were used for quantifying Cu(II) and recording UV-Vis spectra. Fluorescence spectra were obtained using a Hitachi F-4600 fluorescence spectrophotometer from Tokyo, Japan. Images of the aerogels were captured with an iPhone 11 (Apple, Cupertino, CA, USA) using the Colorimeter app (https://www.stellarnet.us/spectroscopy-knowledge-base/stellarpro-software/colorimeter-app/ (accessed on 11 August 2025)) to measure fluorescence intensity.

### 4.3. Preparation of Green Fluorescent C Dots

The process for preparing green fluorescent C dots responsive to Cu(II) ions is outlined as follows. Initially, 1.0 g of D-glucose and 0.80 g of L-aspartate were dissolved in 24 mL of a 0.5 mol/L sodium hydroxide solution. Once fully dissolved, the mixture was transferred to a 50 mL Teflon container and placed in a 150 °C oven to react for 1 h. After the reaction period, the resulting C dot solution underwent dialysis through a membrane with a molecular weight cutoff of 1000 Da for 48 h, with the water being replaced every 3 h. Finally, the C dot solution was pre-frozen and freeze-dried for 48 h with a product yield of 67.4%, after which the dried powder was dissolved in 10 mL of ultrapure water to create a uniform C dot solution.

### 4.4. Preparation of GCDiA via Cross-Linking C Dots and CNC

To prepare the functionalized green C dot-integrated aerogel (GCDiA), cellulose nanocrystals (CNC) were obtained by treating microcrystalline cellulose following a modified protocol. The modification involves using higher NaClO dosage to enhance carboxylation for improved CNC dispersion. Specifically, 0.1 g of TEMPO, 0.5 g of NaBr, and 5.0 g of NaClO were added to a dispersed solution of microcrystalline cellulose (at a mass concentration of 2.5%) and mixed well. Sodium hydroxide was then used to adjust the pH of the solution to 10.0. The resulting precipitate was collected via centrifugation at 6000 rpm after 5 h of shaking and washed six times with deionized water. Subsequently, homogenization was performed using an M110P microfluidizer (Microfluidics International Corporation, Boston, MA, USA) at a pressure of 400 MPa, resulting in CNC with an average length of approximately 200 nm and a diameter of about 5.8 nm.

For the fabrication of GCDiA, various volumes (0.2, 0.4, 0.6, 0.8, 1.0, and 1.2 mL) of the prepared C dot solution were combined with deionized water (DD) to reach a final volume of 2 mL in a 10 mL beaker. Separately, 2 mL of 1%, 2%, 3%, 4%, 5%, and 6% CNC solution in DD were mixed with the C dots solution. To facilitate cross-linking, 0.05 mL of epichlorohydrin (ECH) was added to the mixture. After sealing the container, the combined solutions were incubated at 60 °C for 4 h to ensure sufficient hydrogel formation. The hydrogels were subsequently pre-frozen at −18 °C for 12 h and freeze-dried using a Labconco freeze-dryer (Kansas City, MO, USA) at a pressure below 150 mmHg for 28 h. Based on the CNC concentrations, the resulting aerogels were labeled as GCDiA-1 (1%), GCDiA-2 (2%), GCDiA-3 (3%), GCDiA-4 (4%), GCDiA-5 (5%), and GCDiA-6 (6%). The cylindrical GCDiA structures had a diameter of 2.5 cm and a thickness of 0.8 cm. GCDiA-4 exhibited the strongest fluorescence and was selected for visual monitoring of Cu(II), undergoing comprehensive characterization through Fourier transform infrared spectroscopy (FT-IR), X-ray photoelectron spectroscopy (XPS), and other methods.

### 4.5. Batch Adsorption Experiments

In the batch adsorption experiments, the time-dependent adsorption behaviours of Cu(II) by GCDiA-4 were investigated with initial Cu(II) concentrations of 10, 20, 40, 60, 80, 100, 120, 200, 400, 600, 800, and 1000 mg/L. The weight of GCDiA-4 used for adsorption was 0.20 ± 0.03 g. The amount of Cu(II) adsorbed was determined by indirectly quantifying its concentration in solution using ICP-MS. To assess selectivity, GCDiA-4 was also exposed to the solutions containing various metal ions such as K^+^, Na^+^, Ca^2+^, Ni^2+^, Co^2+^, Cr^6+^, Mg^2+^, Cd^2+^, Cu^2+^, Fe^3+^, and Hg^2+^ at 1000 mg/L. All experiments were performed in triplicate. To quantify the fluorescence of the aerogels, images were captured using a smartphone under UV lamp illumination. The positioning of the UV light, smartphone, and aerogels was standardized to ensure consistent measurement of fluorescence intensity.

### 4.6. Actual Wastewater Adsorption

General wastewater was collected from a printed circuit board factory in Guangdong, with solids in the Cu(II) wastewater pretreated and removed. Water quality parameters were determined based on the weekly average: total COD was 152 mg/L, total Cu was 80.6 mg/L, and pH ranged from 5.3 to 6.0. A uniform sample of 150 mL was taken, filtered through a 22 μm organic filter, and the pH was adjusted to 3 using nitric acid and sodium hydroxide. GCDiA-4 was immersed in the Cu(II) wastewater sample, and its fluorescence was monitored under UV light. Once fluorescence stabilized, the GCDiA-4 was removed, and the liquid it carried was squeezed out. This process was repeated with a second piece of GCDiA-4 until the final piece was added, ensuring that the measurements before and after treatment met the criterion (G_0_ − G)/G_0_ ≤ 1%. The concentration of Cu(II) in the treated sample was calculated, and the color change of the wastewater sample was observed.

## Figures and Tables

**Table 1:** Comparison of the adsorption capacity with many of the reported adsorbent materials.

Material	Ions	Maximum Adsorption (mg/g)	Ref.
Alkaline-modified biomass pectin	Cu(II)	105.00	[59]
Sodium alginate-polyamine adsorbent	Cu(II)	91.00	[60]
Polyacrylamide/chitosan adsorbent	Cu(II)	32.46	[61]
SDS-coated magnetic chitosan	Cu(II)	50.74	[62]
Itaconic acid-grafted magnetite nanocellulose composites	Cu(II)	32.00	[63]
Ionic liquid-modified microcrystalline cellulose Ion-imprinted magnetic chitosan beads	Cu(II) Cu(II)	138.30 78.10	[64] [65]
Nitrogen-doped carbon dots (N-CDs) from carboxymethyl cellulose	Cu(II)	185.00	[66]
Carbon dots@exfoliated layered double hydroxides (CDs@LDH)	Cu(II)	~150.00	[67]
Nitrogen-doped graphene quantum dots (N-GQDs)	Cu(II)	~120.00	[68]
This work	Cu(II)	149.62	/

## Data Availability

The original contributions presented in this study are included in the article. Further inquiries can be directed to the corresponding authors.
